# Rationally Designed Eco‐Friendly Solvent System for High‐Performance, Large‐Area Perovskite Solar Cells and Modules

**DOI:** 10.1002/advs.202300728

**Published:** 2023-05-05

**Authors:** Young Yun Kim, Su‐Mi Bang, Jino Im, Geunjin Kim, Jason J. Yoo, Eun Young Park, Seulki Song, Nam Joong Jeon, Jangwon Seo

**Affiliations:** ^1^ Division of Advanced Materials Korea Research Institute of Chemical Technology (KRICT) 141 Gajeong‐ro Yuseong‐gu Daejeon 34114 Republic of Korea; ^2^ Division of Chemical Platform Technology Korea Research Institute of Chemical Technology (KRICT) 141 Gajeong‐ro Yuseong‐gu Daejeon 34114 Republic of Korea; ^3^ Department of Chemical and Biomolecular Engineering Korea Advanced Institute of Science and Technology (KAIST) 291 Daehak‐ro Yuseong‐gu Daejeon 34141 Republic of Korea; ^4^ Present address: Department of Chemical Engineering and Applied Chemistry Chungnam National University 99 Daehak‐ro Yuseong‐gu Daejeon 34134 Republic of Korea

**Keywords:** eco‐friendly solvents, methylsulfonylmethane, perovskite solar cells, perovskite solar modules

## Abstract

The important but remained issue to be addressed to achieve the mass production of perovskite solar modules include a large‐area fabrication of high‐quality perovskite film with eco‐friendly, viable production methods. Although several efforts are made to achieve large‐area fabrication of perovskite, the development of eco‐friendly solvent system, which is precisely designed to be fit to scale‐up methods are still challenging. Herein, this work develops the eco‐friendly solvent/co‐solvent system to produce a high‐quality perovskite layer with a bathing in eco‐friendly antisolvent. The new co‐solvent/additive, methylsulfonylmethane (MSM), efficiently improves the overall solubility and has a suitable binding strength to the perovskite precursor, resulting in a high‐quality perovskite film with antisolvent bathing method in large area. The resultant perovskite solar cells showed high power conversion efficiency of over 24% (in reverse scan), with a good long‐term stability under continuous light illumination or damp‐heat condition. MSM is also beneficial to produce a perovskite layer at low‐temperature or high‐humidity. MSM‐based solvent system is finally applied to large‐area, resulting in highly efficiency perovskite solar modules with PCE of 19.9% (by aperture) or 21.2% (by active area) in reverse scan. These findings contribute to step forward to a mass production of perovskite solar modules with eco‐friendly way.

## Graphical abstract

A rationally designed eco‐friendly solvent system comprising of GBL and new additive, MSM, is newly suggested. Highly efficient over 24% of PCE and stable (over 1000 h) PSCs are presented. New solvent system is more suitable for mass production of PSCs, achieving ≈21% PCE in 25 cm^2^ solar minimodule, with high tolerance of processing temperature and humidity conditions.

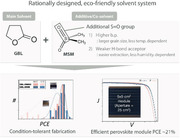

## 1 Introduction

Perovskite solar cells (PSCs) have shown remarkable progress and have achieved a power conversion efficiency (PCE) over 25%, due to their inherent advantages including high absorption coefficient, ambipolar charge transport, long diffusion length, and defect tolerance.^[^ ^1^, ^2^ ^]^ In particular, PSCs can be light‐weight, flexible, and portable with various form factors because they can be fabricated by solution‐process at low temperature and low‐cost, vacuum‐free roll‐to‐roll compatible process.^[^ ^3^, ^4^ ^]^ However, there are still many obstacles to be overcome for mass production of PSCs, of which them is for example, the deposition of high‐quality perovskite films in large‐area by using of eco‐friendly, industry‐compatible solvents excluding dimethylformamide (DMF) as a common processing solvent.

The formation of high‐quality perovskite films in large‐area is challenging yet crucial step to be accomplished. In small‐area, high‐quality perovskite films can be easily obtained via solvent engineering method, employing strongly binding mediator/solvent molecules to retard a rapid and uncontrolled crystallization, and form an intermediate phase, thereby inducing controlled, uniform phase conversion to perovskite photoactive phases by dripping of anti‐solvents during spin‐coating.^[^ ^5^ ^]^ In large‐area and scale‐up fabrication, antisolvent bathing method has been also successfully demonstrated, which was inspired by the solvent‐engineering method in our previous works; in a single web for high‐throughput roll‐to‐roll fabrication, the as‐deposited wet film of the perovskite precursor is continuously immersed into a bath filled with antisolvent to form an intermediate phase and finally produce a photoactive perovskite phase through subsequent drying and thermal annealing step.^[^ ^3^, ^4^ ^]^

The careful selection and design of processing solvent system (including main solvent, co‐solvent/additive and antisolvent) without severe health risk should be necessary for obtaining a high‐quality perovskite film via large‐scale antisolvent bathing method, which requires a usage of a large volume of all processing solvents and a proper ventilation/recycling system. First, the main solvents for dissolution of perovskite precursors should have sufficient solubility to perovskite precursors, and second, antisolvent should have ultimately no solubility to precursors and selective compatibility to the main solvent. To date, a mixture of DMF as the main solvent and dimethyl sulfoxide (DMSO) as a mediator and a co‐solvent/additive was used in most of the previous research works reporting efficient perovskite solar cells. Both solvents have a coordinating ability to lead halide precursors by donating a pair of electrons, and thus have a sufficient solubility over 1 m.^[^ ^6^ ^]^ In addition, a large amount of antisolvent such as toluene, chlorobenzene and diethyl ether have been used for antisolvent dripping and bathing method. However, the current processing solvent system has some limitations considering their environmental impact with a view of industrial mass production, safety, and health hazard dimensions. Moreover, the newly designed solvent system with no toxicity issue should be tolerant to the surrounding environmental atmosphere (e.g., temperature and humidity) during the fabrication process.

DMF is known to have serious health issues such as hepatotoxicity, reproductive toxicity and skin/eye irritation, so only limited amount is allowed to be used in industrial processes.^[^ ^7^, ^8^, ^9^ ^]^ DMSO is relatively benign for human health, but it can induce the percutaneous absorption of Pb ions by mixing with water. In addition, DMSO has relatively low melting point (≈18 °C), so it is difficult to perform a processing with DMSO at a low‐temperature environment. Furthermore, a strongly binding nature of DMSO to perovskite precursor have rather adverse effect on extraction of mediator molecule, that is, DMSO, by antisolvent bathing method in large‐area, because there is no external physical forces applied during bathing process.^[^ ^10^, ^11^ ^]^

There have been several research works reported to replace DMF:DMSO solvent system with new solvents. Acetonitrile have been selected as an alternative, but it necessitates additional components to boost solubility such as methylamine gas,^[^ ^12^ ^]^ tetrahydrofuran,^[^ ^13^ ^]^ or 2‐methoxyethanol,^[^ ^14^ ^]^ which are also harmful to human health. In addition, their usage is only limited to methylammonium lead iodide, which exhibits relatively lower efficiency and poor stability. 2‐butoxyethanol (2‐BE) has been also used, but it requires 90 v/v % of DMSO co‐solvent due to limited solubility of 2‐BE, which is difficult to completely remove high volume percentage of DMSO with high boiling point after bathing or annealing.^[^ ^15^ ^]^ Recently, the usage of ethyl alcohol (EtOH) mixed with alkyl‐amines and dimethylacetamide (DMAc) has been reported as a green solvent system, but the effect of volatile alkyl amine is less considered, which is skin irritant, and DMAc is also calcinogenic.^[^ ^16^ ^]^

Gammabutyrolactone (GBL) is relatively eco‐friendly and free from any processing limitations such as melting point.^[^ ^17^ ^]^ GBL have been widely used in early works of PSCs,^[^ ^5^ ^]^ but rarely used for formamidinium lead iodide (FAPbI_3_)‐based mixed cation and halide system, mainly due to its relatively low solubility. In order to obtain a high‐quality perovskite film processed from GBL, careful selection of co‐solvent or additive is required. New co‐solvent/additive should have a high solubility to the precursor and should be eco‐friendly with suitable melting/boiling point. Gardner et al., reported a new solvent system is composed of GBL, EtOH and acetic acid (AA), where selective solubility of EtOH to organic halide molecules can be problematic, and residual AA can deteriorate perovskite layer.^[^ ^6^ ^]^

To address several issues as discussed above, we carefully choose methylsulfonylmethane (MSM) as an eco‐friendly co‐solvent/additive to GBL solvent. With a respect to molecular structure, MSM has extra S=O group in addition to DMSO. It can be expected to have a suitable binding affinity to perovskite precursor, which is capable of enhancing overall solubility of the precursors in the mixture of GBL and also controlling the activation energy to form the solvate‐intermediate phase with the precursors. Moreover, additional polar group of S=O into DMSO can affect a physical property of DMSO solvent; MSM has much higher melting point and boiling point than those of DMSO. These properties are essential for determining the evaporation rate during the coating and the thermal annealing step of the perovskite layer, which has a strong impact on the crystallization of the perovskites involving the nucleation and the growth. It is also noteworthy that MSM is a very safe chemical in terms of human health, even can be used as a medical supplement.

In this work, an eco‐friendly solvent system is newly designed for fabrication of perovskite layer via antisolvent bathing in both small and large area. Not only the solvent of precursor is substituted to eco‐friendly solvents, that is, GBL and MSM, but the antisolvent is also selected to eco‐friendly one, namely, butyl acetate (BA). The physical properties of newly introduced MSM are systematically investigated. A better quality of perovskite films can be achieved by using GBL as main solvent with MSM as a co‐solvent/additives and BA as an antisolvent, thereby resulting in high‐performance, small‐area PSCs having a PCE of 24% (in reverse scan), with remarkable stability for 1000 h under continuous light‐soaking and damp‐heat tests. Notably, MSM additives are also beneficial to produce PSCs in low‐temperature or high‐humidity conditions, proven by improved device performances compared to PSCs based on DMSO. The feasibility of newly developed, eco‐friendly solvent system for large‐area production of perovskite layer is also confirmed. Finally, large‐area perovskite solar modules with aperture size of 25 cm^2^ are fabricated, yielding PCEs of 19.9% and 21.2% in reverse scan, from aperture area and active area, respectively.

## 2 Results and Discussion

An eco‐friendly solvent system is newly designed to be capable of a sufficient solubility for the perovskite precursors and a suitable processibility for antisolvent bathing method. MSM is introduced as a co‐solvent/additive for GBL as a main solvent. MSM is an organic molecule, which has an extra S=O functional group than DMSO. Due to a higher molecular weight and more polar group of MSM than DMSO, MSM has a much higher melting and boiling point of 109 and 248 °C, respectively, than those of DMSO (melting and boiling point of 19 and 189 °C, respectively). In contrast, the bifurcated oxygen of MSM could increase oxidation state of the sulfur, reduce the Mulliken charge density on oxygen, and donor number as compared with DMSO.^[^ ^18^ ^]^ Therefore, MSM is expected to have a weaker interaction with perovskite precursor than DMSO, which is widely used as strongly binding co‐solvent/mediator.

**Figure 1:**
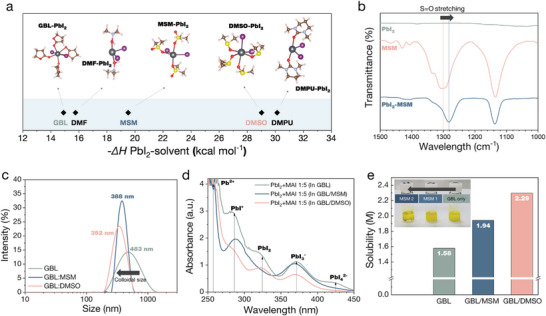
a) Enthalpy change (−ΔH PbI_2_) of various solvents with PbI_2_ for adduct formation by a DFT calculation based on optimized molecular structures. b) FT‐IR spectra of pure PbI_2_, MSM, and PbI_2_‐MSM powders. c) DLS measurements of colloidal dispersions dissolved in GBL, GBL:DMSO, and GBL:MSM. d) UV–vis absorption spectra of PbI_2_/MAI solutions with GBL, GBL:DMSO, and GBL:MSM, e) Solubility of PbI_2_ in GBL, GBL:MSM, and GBL:DMSO.

Binding strength of various solvent molecules including MSM with PbI_2_ are compared by density functional theory (DFT) calculation of the enthalpy change of molecules for solvation of PbI_2_.^[^ ^19^ ^]^ (**Figure** **1**a) The number and configuration of solvents for solvation of PbI_2_ are optimized and given in Figure 1a. From the calculation, GBL, known as a non‐coordinating solvent, showed the lowest binding energy with PbI_2_, while DMSO and DMPU (*N*,*N*′‐Dimethylpropyleneurea), known as strongly coordinating solvents showed higher binding energy. MSM has a moderate binding energy of in between GBL and DMSO, which could be beneficial for providing a proper solubility for perovskite precursor and a suitable environment to be easily extracted by antisolvent bathing at the same time.

MSM molecules are capable of effectively interacting with perovskite and PbI_2_. Binding of MSM to PbI_2_ is clearly validated by Fourier transform infrared (FT‐IR) spectra shown in Figure 1b. The characteristic peak assigned to S=O stretching of MSM shifts from 1301 to 1282 cm^−1^, indicating a molecular interaction between MSM and PbI_2_ by electron donating from S=O functional group of MSM to PbI_2_. MSM also can interact with FAI molecules, which is confirmed by the shift of C=N stretching peak of FAI from 1691 to 1695 cm^−1^ in FT‐IR spectra.^[^ ^20^ ^]^ (Figure S1, Supporting Information) Such an interaction of MSM with both FAI and PbI_2_ molecules can cause the efficient solvation/binding of MSM to perovskite precursors.

As discussed above, MSM showed intermediate binding affinity to perovskite precursor and PbI_2_, stronger than GBL but weaker than DMSO. Solvation strength of each solvent is compared by dynamic light scattering (DLS) spectra of perovskite solution. (Figure 1c) Median values of peak of GBL, GBL:MSM and GBL:DMSO are decreased from 483, 377 to 352 nm, respectively. Stronger binding affinity between solvents and the perovskite precursors can effectively reduce surface energy, thereby forming smaller stable colloids in the solution. The solvation strength is also compared by UV–visible (UV–vis) spectra of PbI_2_+MAI dilutions in various solvents. (Figure 1d) To bind with center Pb atoms and form a plumbate octahedra, solvent molecules should compete with I^−^ ions. Stronger affinity of solvent to Pb atoms leads to more substitution of I^−^ ions to solvent molecules, thereby resulting in a presence of lower‐order solvated iodoplumbate species.^[^ ^21^ ^]^ For the sample in GBL, all of lower and higher order iodoplumbates are present in the solution, but lower‐ order species are dominant as for the MSM and DMSO samples. Specifically, lower‐order species are more dominant in the solution of GBL:DMSO than that of GBL:MSM. The solubility of perovskite precursor for GBL, GBL:MSM and GBL:DMSO are measured by directly dissolving oprecursors into solvents. (Figure 1e) By adding MSM into GBL, the solubility is improved from 1.58 to 1.94 m, whereas the solubility is then further improved up to 2.29 m by dissolving precursors in GBL:DMSO. From inset of Figure 1e, adding more amount of MSM into GBL results in the increase of the solubility, indicating that MSM acts as an effective solvation molecule.

**Figure 2:**
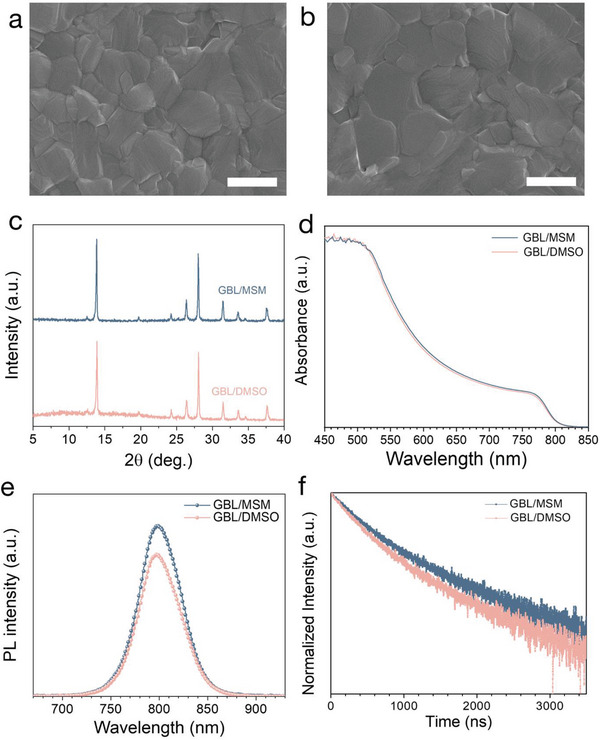
Top‐view SEM images of the perovskite films fabricated from a) GBL/DMSO, and b) GBL/MSM solvents. Scale bar is 1 µm. c) XRD spectra, d) absorption spectra, e) photoluminescence spectra, and f) time‐resolved photoluminescence spectra of the perovskite films fabricated from GBL:DMSO and GBL:MSM solvents.

We investigated a function of MSM as a mediator and a co‐solvent/additive to retard a fast crystallization of the precursors, form an intermediate phase and be easily removed by antisolvent bathing and thermal annealing during the preparation of the perovskite film. The perovskite films fabricated by GBL:MSM solutions showed better quality than that made from GBL:DMSO. All the films are fabricated by spin‐coating of perovskite solution dissolved in GBL:MSM or GBL:DMSO, followed by subsequent bathing in eco‐friendly antisolvent, BA. The effect of using DMSO and MSM as mediator solvent on the morphological aspect of perovskite films is investigated by analyzing the top‐view field emission scanning electron microscope (FE‐SEM) images. (**Figure** **2**a and b) As shown in Figure 2a,b, perovskite films made from both GBL:MSM and GBL:DMSO show dense and pin‐hole‐free morphologies. Grain size was measured by using ImageJ software, and individual size of grains are provided in Table S1, Supporting Information. Notably, the MSM‐based film has larger crystal grain size (with 1065 ± 268 nm) as compared with that (769 ± 244 nm) of the DMSO‐based film. The increase in grain size of MSM‐based perovskite film is mainly attributed to the higher boiling point of MSM despite of weaker binding energy with PbI_2_, which can reduce the number of nuclei at the supersaturation state of the intermediate phase induced just after antisolvent bathing and initial annealing, thereby giving rise to form enlarged‐grains during further annealing process. Uniformity of perovskite film made by using GBL:MSM was confirmed by low‐magnification SEM image. (Figure S2, Supporting Information)

The perovskite films made from MSM exhibit almost identical crystallographic and optical properties to that from DMSO. In X‐ray diffraction (XRD) spectra, both perovskite films show similar peak intensity at 13.8°, which is assigned to (001) planes of (FAPbI_3_)_0.95_(MAPbBr_3_)_0.05_. (Figure 2c) Moreover, the perovskite films from the both solvents show similar ultraviolet‐visible (UV–vis) absorption spectra. (Figure 2d)

We also examine the effect of antisolvent bathing and dripping methods on the film fabrication by FE‐SEM and XRD measurements. (Figures S3 and S4, Supporting Information) Both films were fabricated from the GBL:MSM solutions with BA antisolvent. In SEM images, average grain size (end‐to‐end distance) were 1104 ± 364 and 1214 ± 330 nm for the film fabricated by dripping and bathing, respectively. In Figure S4, Supporting Information, perovskite film showed much improved intensity of (001) peak for the sample produced by bathing. Overall, antisolvent bathing process using GBL:MSM in this work is advantageous for producing more crystalline film with larger grain size and reducing defective grain boundaries, as compared to dripping process.

To gain more understanding of charge recombination dynamics, we further measured photo‐luminescence (PL) and time‐resolved photoluminescence (TRPL) spectra of the perovskite films processed from GBL:DMSO and GBL:MSM. (Figure 2e,f) The steady‐state PL intensity of the perovskite film processed with GBL:MSM is enhanced ≈20% compared to the GBL/DMSO‐based perovskite film, without any changes in maximum emission wavelength (795 nm). The TRPL decay curves were fitted by bi‐exponential decay model using a fast (*τ*_1_) and a slow (*τ*_2_) decay processes. Previous reports suggested that fast decay is mainly related to trapped free carriers from neighboring interlayers to perovskite film, and the slow decay is dominated to the charge recombination in the perovskite film.^[^ ^22^ ^]^ The perovskite film fabricated by GBL:MSM showed the average lifetime of 530 ns, while the GBL:DMSO film shows a reduced lifetime of 432 ns.

The high‐quality perovskite film made from GBL:MSM than that from GBL:DMSO was confirmed by SCLC measurement in hole‐only device. (Figure S5, Supporting Information) In *a J–V* curve, trap‐filled‐limit voltage (*V*_TFL_) for GBL:MSM sample is 1.26 V, which is reduced from the value for GBL:DMSO sample, 1.49 V. V_TFL_ is related with density of defects, which is described by following equation: $V_{\mathrm{TFL}}=\frac{qn_{t}L^{2}}{2\varepsilon }\;$, where q is elementary charge, n_t_ is the trap density, L is thickness, and *ε* is dielectric constant.^[^ ^23^ ^]^ Based on the formula and values given in previous literature, trap density for GBL:MSM sample is 5.44 × 10^16^ cm^−3^, which is smaller than that of GBL:DMSO sample, which is 6.44 × 10^16^ cm^−3^.

In order to understand such an enhanced carrier lifetime of the perovskite film obtained from GBL:MSM, we also explore the defects at the surface of the perovskite film by X‐ray photoelectron spectroscopy (XPS) analysis. (Figure S6, Supporting Information) Interestingly, a significant shift of Pb 4f, I 3d, and N 1s peaks in XPS spectra of the sample from GBL:MSM is observed as compared to that of the sample from GBL:DMSO. It implies a reduction of the surface defects, which can occur, probably due to an unexpected passivation effect of the residual MSM molecules in the perovskite film. As a result, we achieved a better crystalline perovskite film with more enlarged grains and less defects when we replaced GBL:DMSO with GBL:MSM. Perovskite samples fabricated using GBL:MSM and GBL:DMSO were also analyzed for their S 2p XPS spectra. (Figure S7, Supporting Information) There is a small peak at 161.5 eV for the GBL:DMSO sample, whereas the GBL:MSM sample has no discernible peak. According to a previous report, this peak may originate from residual DMSO in the final perovskite film, which can be problematic for the long‐term operation of PSCs.^[^ ^10^ ^]^

MSM is also crucial for completely conversion of precursors to *α*‐phase of FA‐based perovskite film. Without methylammonium chloride (MACl), we fabricated as‐prepared films using GBL:MSM and GBL:DMSO after antisolvent bathing, prior to thermal annealing. For GBL:MSM, the perovskite film exhibited only a strong (001) *α*‐phase peak (at ≈13.8°) of FAPbI_3_ in XRD spectra (as shown in Figure S8, Supporting Information). However, for GBL:DMSO, two main peaks are observed, corresponding to PbI_2_ and *α*‐phase of FAPbI_3_. Previous studies showed that perovskite film without MACl results in a presence of strong PbI_2_ peak for annealed films, originated from an incomplete conversion of precursor to perovskite phase.^[^ ^24^ ^]^ Both films showed similar morphology and grain sizes from SEM images (Figure S9, Supporting Information), but using MSM would induces *α*‐phase of FAPbI_3_, which is expected to beneficial to retain phase stability during operation.

**Figure 3:**
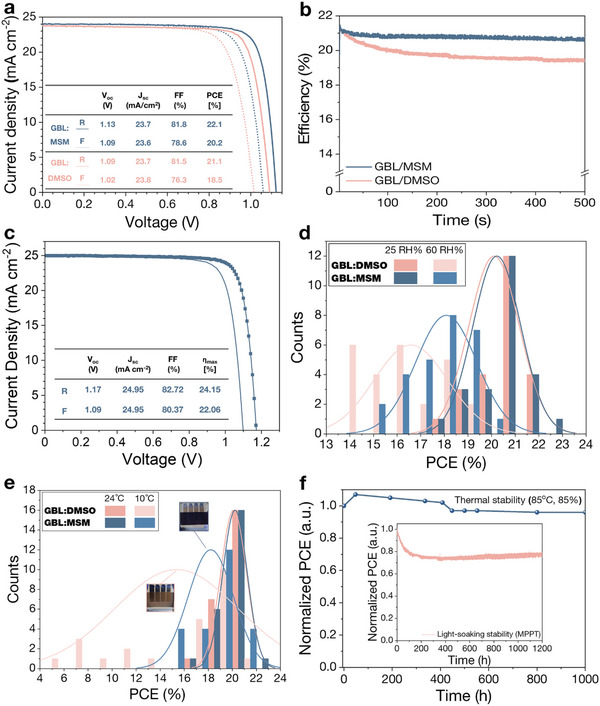
a) Representative *J–V* curves and b) MPPT measurement of perovskite solar cells (PSCs) fabricated by using GBL:DMSO and GBL:MSM solvents. c) Champion *J–V* curves of GBL:MSM‐based PSC. d) PCE distributions of PSCs fabricated by using GBL/DMSO and GBL/MSM under d) 25 and 60 RH%, and e) 10 and 24 °C. f) Normalized PCE of PSCs after damp heat test (85 °C, 85 RH%) and continuous light‐soaking test.

**Figure** **3**a shows representative current density‐voltage (*J–V*) curves of the PSCs fabricated with GBL:MSM and GBL:DMSO. All the PSCs were fabricated under ambient atmosphere (humidity of 20%–25% and temperature at 20 °C). PSCs fabricated by GBL:MSM exhibit higher PCE (22.1% and 20.2% from reverse scan (RS) and forward scan (FS), respectively), while the PSCs fabricated by GBL:DMSO show lower PCE (21.1% from RS and 18.5% from FS, respectively). The short‐term device stability was monitored by a maximum power‐point tracking (MPPT) measurement under AM 1.5G illumination at ambient air condition. (Figure 3b). GBL:MSM‐based device shows more stable and higher power output than that from GBL:DMSO, yielding a stabilized PCE of 20.7%. For GBL:MSM, we optimized the device fabrication by applying the surface passivation of hexylammonium iodide, yielding a champion PCE of 24.15% in reverse scan and 22.06% in forward scan. (Figure 3c) External quantum efficiency (EQE) curve shows effective light absorption of perovskite film made by using GBL:MSM over the whole visible range. (Figure S10, Supporting Information) *J*_SC_ value calculated by convolution of the EQE curve for AM1.5G irradiation spectra is 24.9 mA cm^−2^, which is well‐matched with *J*_SC_ obtained from the *J–V* curve. We varied the amount of MSM in the solution to set the optimum condition. The resulting PSCs showed very similar performance regardless of the amount of MSM added in the precursor solution. We set the default condition as 1 eq. molar amount of MSM with respect to the perovskite. (Figure S11, Supporting Information) The improved performance achieved by substituting MSM for DMSO was also confirmed using DMF as the main solvent. (Figure S12, Supporting Information) This may be due to the larger grain size observed in SEM images for the DMF:MSM system as compared to the DMF:DMSO system.

GBL:MSM solvent system showed a wider processing window under various humidity and temperature. The PCE distribution of PSCs fabricated under low (25 RH%) and high (60 RH%) humidity is depicted in Figure 3d. The performance of PSCs is decreased under high humidity condition, but GBL:MSM‐based PSCs showed a relatively smaller decrease in PCE under 60% RH than GBL:DMSO‐based PSCs. MSM has limited solubility in water compared to DMSO, and additional S=O bonding of MSM results in lower Mulliken charge density in oxygen, thereby leading to weaker acceptance for hydrogen bonding from water molecules. Overall, the energy of interaction for solvents with water is higher for DMSO than MSM, as reported previously, resulting in a lower amount of H_2_O in intermediate phase.^[^ ^18^ ^]^ In previous work, water molecules tend to participate in the intermediate phase such as PbI_2_‐FAI‐H_2_O‐DMSO, and facilitate more rapid phase transformation.^[^ ^25^ ^]^ Therefore, perovskite wet‐film with higher water contents tends to form more irregular film, and degraded performance in resulting PSCs. The perovskite films fabricated with GBL:MSM and GBL:DMSO processed at different humidity are compared also by SEM images and XRD spectra. (Figures S13 and S14, Supporting Information) The films fabricated under high humidity showed more rough surfaces and reduced crystallinity. However, the crystallinity of the film fabricated under high humidity is much higher for GBL:MSM than GBL:DMSO, validating a wider processing window of GBL:MSM under humidity.

Figure 3e showed PCE distribution of PSCs under various temperatures (10 and 25 °C). At ambient processing temperature (25 °C), both GBL:MSM and GBL:DMSO‐based PSCs showed narrow PCE distribution with a high average PCE of ≈20%. For the PSCs with processing temperature of 10 °C, however, GBL:MSM‐based PSCs showed much higher average PCE with narrow PCE variations, compared to GBL:DMSO‐based PSCs. DMSO has a melting point of 18 °C, so it is likely that DMSO does not work effectively as a mediator at low temperature. In contrast, MSM has a high melting point of 109 °C and lower binding energy with PbI_2_, therefore its function as a mediator is less susceptible to the processing temperature. The perovskite films fabricated with GBL:MSM and GBL:DMSO processed at different temperatures are compared also by SEM images and XRD spectra. (Figures S15 and S16, Supporting Information) In SEM images, both films showed negligible changes in average grain size, but GBL:DMSO‐based perovskite film showed many pin‐holes and lower coverage when it is processed at 10 °C. In XRD spectra, perovskite film processed by GBL:DMSO showed decrease in its crystallinity when it is processed at 10 °C. These morphological and crystallographic aspects are thought to be relevant to the decrease in the performance of GBL/DMSO‐devices processed at 10 °C.

The PSCs fabricated by using GBL:MSM as a solvent showed superior device stability under various external stresses including continuous light‐soaking, thermal and humid stress. The long‐term stability measurements of PSCs based on GBL:DMSO and GBL:MSM were conducted under continuous light soaking and thermal stress as shown in Figure 3f. During the light‐soaking stability test, PSCs were encapsulated and kept at the maximum power‐point under 1‐sun condition (100 mW cm^−2^, AM 1.5G) at ≈45 °C. The device fabricated by GBL:MSM demonstrated outstanding light‐stability, maintaining more than 78% of the initial performance even after 1200 h. We also conducted a damp heat test under 85 °C/85% RH condition to evaluate the stability of encapsulated devices under thermal and humid stress. For the damp‐heat test, poly(triaryl amine) (PTAA) is used as a hole‐transporting layer. Representing *J–V* curves of GBL:MSM and GBL:DMSO‐based devices with PTAA are shown in Figure S17, Supporting Information. The device with GBL:MSM kept over 96% of its initial PCE after 1000 h, with a slight decrease of short‐circuit current density (*J*_SC_) and fill factor (FF).

**Figure 4:**
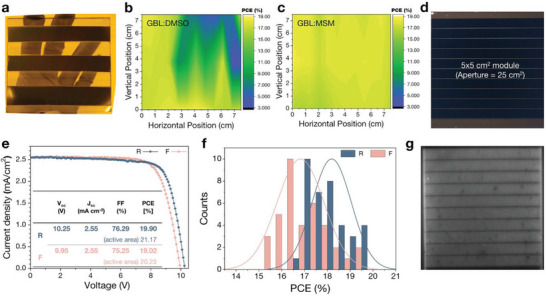
a) Large‐area (5 × 5 cm^2^) perovskite films processed with eco‐friendly solvent/antisolvent and PCE distribution of resulting devices made by using b) GBL:DMSO and c) GBL:MSM solvents. d) The optical image of perovskite solar module fabricated by using eco‐friendly solvent system. e) *J–V* curves of champion module. f) Histogram of perovskite solar modules. g) The electroluminescent image of perovskite solar module.

Furthermore, we extended our approach employing a newly developed eco‐friendly solvent system into large‐area fabrication. All the constituent layers of PSCs were deposited on a 7 × 7 cm^2^ substrate with the patterned ITO, then the large‐area substrate was divided into 9 unit cells by cutting the substrate. Finally, their efficiencies were measured for each cell. (**Figure** **4**a‐c and Figure S18, Supporting Information) PSCs fabricated by GBL:MSM showed superior reproducibility from cell to cell, as compared to those fabricated by GBL:DMSO. This behavior was supported by the following observations; The perovskite films obtained from large‐area fabrication using GBL:MSM showed similar quality to the unit cell, with respect to morphological aspect, as shown in Figure S19, Supporting Information. These films also showed high uniformity in thickness, as shown in Figure S20, Supporting Information.

For a practical application of our newly developed eco‐friendly solvent system employing antisolvent bathing, we fabricated a perovskite solar sub‐module with the 7 × 7 cm^2^ substrate, consisting of the nine sub‐cells in series. The aperture area of the module is 25 cm^2^, as shown in Figure 4d. The champion *J–V* curve of perovskite sub‐module shows a high PCE of 19.90% (from aperture area) under RS scan, with an open‐circuit voltage (*V*_OC_) of 10.25 V, a *J*_SC_ of 2.55 mA cm^−2^, and FF of 76.29%, and a high PCE of 19.02% under FS scan, with a *V*_OC_ of 9.95 V, a *J*_SC_ of 2.55 mA cm^−2^, and FF of 75.25%, respectively. (Figure 4e). The performance calculate active area corresponds to 21.17% and 20.23% under RS and FS scan, respectively. The statistical distribution of the PCEs recorded for the fabricated 36 sub‐modules is depicted in Figure 4f. Average PCE of perovskite solar modules is estimated to be 18.18% in RS and 16.8% in FS. Electroluminescence (EL) intensity of large‐area perovskite solar module was measured to confirm the uniformity of the 7 × 7 cm^2^ module as shown in Figure 4g, Supporting Information. The EL image shows the influence of the electrical losses, revealing the distribution of the induced photocurrent in‐line within the fabricated module.^[^ ^26^, ^27^ ^]^ The EL image of the perovskite module clearly shows uniform intensity variation throughout whole area.

In conclusion, the eco‐friendly solvent system is newly developed for fabrication of high‐performance solar cells and modules via an antisolvent bathing method. The new solvent system is designed to have sufficient solubility and proper binding strength to perovskite precursors to be efficiently extracted by bathing method, and the results are confirmed by a DFT calculation and systematic characterizations of physical/chemical properties. A perovskite film fabricated by deposition from GBL:MSM and subsequent bathing in eco‐friendly antisolvent, BA exhibited a better quality than that from GBL:DMSO, thereby resulting in a high‐performance, small‐area PSCs having a PCE of over 24% (in reverse scan). Moreover, the resulting PSCs showed good stability over 1000 h under continuous light soaking or damp‐heat conditions. In addition, using the GBL:MSM solvent system was advantageous for producing PSCs at low temperature (10 °C) or high humidity (60 RH%) compared to GBL:DMSO system. The eco‐friendly solvent system was finally applied to the fabrication of large‐area films and 7 × 7 cm^2^ perovskite solar modules, leading to high PCE up to 19.9% (by aperture) or 21.2% (by active area) in reverse scan. The newly designed, eco‐friendly solvent system suggested in this study would pave a new way to commercialize the large‐area perovskite solar modules in a more eco‐friendly and efficient way.

## 3 Experimental Section

### Materials

All chemicals were purchased from Sigma‐Aldrich, Alfa Aesar, or GreatCellSolar unless specified and used as received.

### Preparation of Perovskite Precursor Solutions

A perovskite solutions were prepared by dissolution of 2 m of formamidinium lead triiodide (FAPbI_3_) with 5 mol% of methylammonium lead tribromide (MAPbBr_3_) and 35 mol% of methylamine hydrochloride (MACl) in *γ*‐Butyrolactone (GBL)/dimethyl sulfoxide (DMSO) or methyl sulfonyl methane (MSM) at a ratio of 8:1, respectively.

### Fabrication of Perovskite Solar Cells

Perovskite solar cells were fabricated with the configuration of fluorine‐doped tin‐oxide (FTO) (Pilkington, TEC8)/blocking TiO_2_ layer (bl‐TiO_2_)/mesoporous TiO_2_ (mp‐TiO_2_)/Sodium bis(trifluoromethylsulfonyl)imide (Na‐TFSI)/perovskite/Spiro‐OMeTAD/Au.^[^ ^28^ ^]^ First, Pre‐patterned FTO glass substrates were sequentially cleaned by detergent, DI water, ethyl alcohol, acetone and 2‐propanol. The bl‐TiO_2_ was coated onto a FTO substrate by spray pyrolysis at 450 °C using a titanium diisopropoxide bis(acetylacetonate) dissolved in ethanol. Subsequently, the mp‐TiO_2_ (Sharechem) paste diluted in mixed solvent of 2‐methoxyethanol and terpineol (3.5:1, w/w) was spin‐coated onto FTO substrate/bl‐TiO_2_ and heated at 500 °C for 1 h. After the heat treatment of mp‐TiO_2_, the Na‐TFSI, (31.6 mg mL^−1^ in acetronitrile) was spin‐coated and then annealed at 500 °C for 30 min. Perovskite layer was deposited by spin‐coating onto FTO substrate/bl‐TiO_2_/mp‐TiO_2_ with perovskite solution. After spin‐coating, substrate was dipped in butyl acetate (BA, Sigam‐Aldrich) as an anti‐solvent and air was blown onto substrate in order to remove excess BA from the film. Then, the intermediate perovskite film was annealed at 150 °C for 10 min. After that, Spiro‐OMeTAD (LumTec) was dissolved in chlorobenzene (0.091 g mL^−1^) with 21 µL of bis‐(trifluoromethane)sulfonimide lithium salt (Li‐TFSI, Sigma‐Aldrich) solution (540 mg mL^−1^ in acetronitrile), 35 µL of 4‐*tert*‐butylpyridine (tBP, Sigma‐Aldrich) and 9 µL of tris(2‐(1H‐pyrazol‐1‐yl)‐4‐tert‐butylpyridine)cobalt(III) tri[bis(trifluoromethane)sulfonamide] (FK209, (LumTec) solution (376 mg mL^−1^ in acetronitrile). The Spiro‐OMeTAD film was spin‐coated at 2000 rpm for 30 s. Finally, Au electrode (≈70 nm) was formed by thermal evaporation in a vacuum of 1 × 10^−6^ torr.

### Fabrication of Perovskite Solar Modules

The perovskite solar module composed of nine stripes in series on 7 × 7 cm^2^ substrate were patterned by CO_2_ laser (EL‐MKRF Series, South Korea) with a power of 85 W. For the solar module, this work designed the module to obtain a geometric fill factor (GFF) of 94.4% and each P1 lines were patterned by scribing width of 100 µm to separate the FTO substrate. Next, bl‐TiO_2_/mp‐TiO_2_/perovskite/Spiro‐OMeTAD layers were coated and P2 lines with a width of 100 µm to expose the bottom FTO substrate to connect the series linkages between cells. Finally, Au electrodes with a thickness of ≈70 nm was then formed by thermal evaporation and each submodules were separated by laser scribing to form P3 lines.

### Characterization of Devices

The morphology of the perovskite film with GBL:DMSO or GBL:MSM was obtained by field emission scanning electron microscope (FE‐SEM, Mira 3 LMU FEG, Tescan). To identify the crystal phase of the prepared perovskite films, the X‐ray diffraction (XRD, Smart Lab, Rigaku) spectra were obtained by an X‐ray diffractometer from 5 to 45 degree. The absorption spectra were obtained in the wavelength range of 300 to 900 nm by using an UV–vis spectrophotometer (UV‐2550, Shimadzu). To evaluate the device performances, *J–V* characteristics were measured under simulated standard conditions (100 mA cm^−1^, AM 1.5 G illumination) by using a solar simulator (Oriel Class A, 91195A, Newport) with a voltage source meter (Keithley 2420 Instruments, USA). All devices were masked with metal mask having a fixed active area (0.094 cm^2^). For hole‐only device, NiOx nanoparticles (2.5 wt% in IPA, Avantama Ltd.,) was first deposited on the top of ITO/glass substrate, then MeO‐4PACz, perovskite layer and Spiro‐OMeTAD layer was sequentially deposited by spin‐coating and thermal annealing. Finally, Au electrode was formed. The transient photoluminescent (TRPL) decay curves obtained on an optical parametric oscillator (OPO) laser system (NT 342A‐10‐AW, EKSPLA) with a 532 nm excitation wavelength (pulse energy of ≈1 µJ). The emitted photoluminescence (PL) was collected by a monochromator (SP2150, Princeton Instruments) detected with a photomultiplier tube (PMT, Hamamatsu, H10721‐20). The output signal was recorded using a digital oscilloscope (DSO‐X 3054A, Agilent). Fourier‐transform infrared (FT‐IR) spectra were measured by Bruker Alpha‐p with ATR mode. X‐ray photoelectron spectroscopy (XPS) spectra was measured by using multi‐purpose XPS spectrophotometer (Sigma Probe). The dynamic light scattering (DLS) measurement was conducted by particle size analyzer (Anton Paar Litesizer 500). Electroluminescent (EL) image of the module was obtained by using solar cell imaging test system (K3300, McScience) with a power supplier.

### Device Stability

Long‐term photo‐stability of the devices were performed by MPPT with the source meter (Keithley 2420, USA). A 450 W xenon lamp (Oriel, USA) with an AM 1.5G filter was used as a light source in an air chamber. The device temperature and humidity in the chamber were maintained at about 45 °C and 25% RH, respectively. Thermal stability testing was performed with encapsulation in a thermo‐hygrostat (TH‐DG‐150, JEIO TECH) under a humid environment about 85% RH at 85 °C.

## Conflict of Interest

The authors declare no conflict of interest.

## Supporting information

Supporting Information

## Data Availability

The data that support the findings of this study are available from the corresponding author upon reasonable request.
