# Supplementary material for: Rationally Designed Eco‐Friendly Solvent System for High‐Performance, Large‐Area Perovskite Solar Cells and Modules
Source: Adv Sci (Weinh). 2023 May 5;10(20):2300728. doi: 10.1002/advs.202300728 (PMC10369249; doi:10.1002/advs.202300728)
Supplement: Supplementary file 1 — Supporting Information [file ADVS-10-2300728-s001.pdf]

ADVANCED SCIENCE
Open Access

Supporting Information

for *Adv. Sci.*, DOI 10.1002/advs.202300728

Rationally Designed Eco-Friendly Solvent System for High-Performance, Large-Area Perovskite Solar Cells and Modules

*Young Yun Kim, Su-Mi Bang, Jino Im, Geunjin Kim, Jason J. Yoo, Eun Young Park, Seulki Song, Nam Joong Jeon and Jangwon Seo**

[Supplementary Information]

Rationally Designed Eco-friendly Solvent System for Highly Efficient, Large-area Perovskite Solar Cells and Modules

Young Yun Kim[†], Su-Mi Bang[†], Jino Im, Geunjin Kim, Jason J. Yoo, Eun Young Park, Seulki Song[¶], Nam Joong Jeon, Jangwon Seo*

Dr. Y. Y. Kim, S.M. Bang, Dr. G. Kim, Dr. J. J. Yoo, E. Y. Park, Dr. S. Song, Dr. N. J. Jeon, Dr. J. Seo

Division of Advanced Materials, Korea Research Institute of Chemical Technology (KRICT), 141 Gajeong-ro, Yuseong-gu, Daejeon 34114, Republic of Korea

Dr. J. Im

Division of Chemical Platform Technology, Korea Research Institute of Chemical Technology (KRICT), 141 Gajeong-ro, Yuseong-gu, Daejeon 34114, Republic of Korea

Dr. J. Seo

Department of Chemical and Biomolecular Engineering, Korea Advanced Institute of Science and Technology (KAIST), 291 Daehak-ro, Yuseong-gu, Daejeon 34141, Republic of Korea

E-mail: jwseo@kaist.ac.kr

† Those authors are equally contributed.

¶ Present address: Department of Chemical Engineering and Applied Chemistry, Chungnam National University, 99 Daehak-ro, Yuseong-gu, Daejeon, 34134 Republic of Korea

Keywords: perovskite solar cells, methylsulfonylmethane, eco-friendly solvents, perovskite solar modules

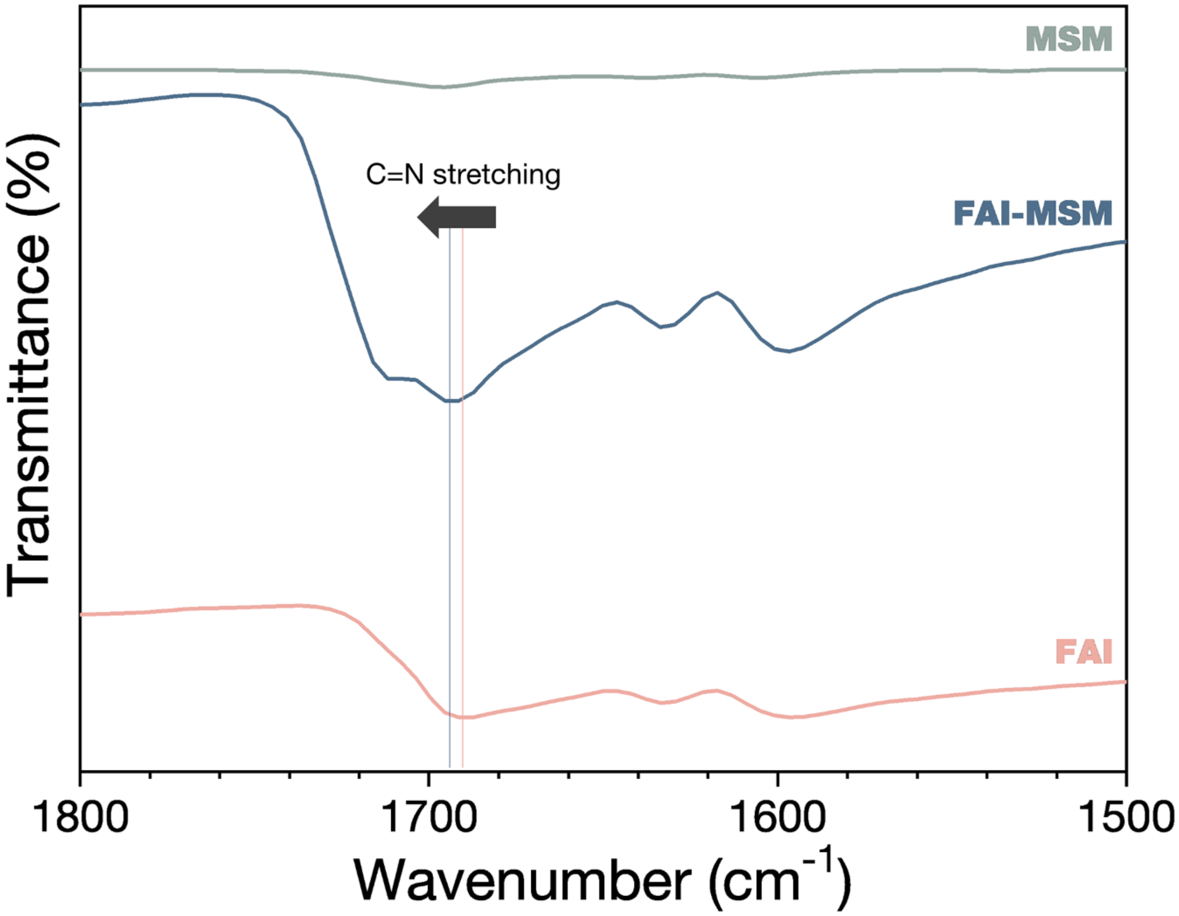

Figure S1. FT-IR spectra of FAI, FAI-MSM, and MSM powders.

Table S1. Measured size of grains for perovskite film made by using GBL:MSM and GBL:DMSO

Index	GBL:DMSO	GBL:MSM
1	484.32	1064.336
2	685.733	1258.503
3	713.477	1264.015
4	757.161	798.506
5	1063.438	1005.764
6	492.043	1259.932
7	845.14	701.155
8	556.345	1291.711
9	512.301	1109.925
10	1097.788	886.896
11	847.94	1306.995
12	514.199	1327.986
13	528.493	933.939
14	939.598	765.318
15	851.131	694.226
16	1167.655	1398.854
17	921.334	1604.646
18	406.926	1438.343
19	924.968	695.089
20	1054.65	731.941
21	1127.899	1191.126
22	505.386	732.517
23	491.396	931.531
24	1056.34	1099.12
25	678.672	1152.37
**Mean**	**768.9733**	**1065.79**
**SD**	**244.0714**	**267.8738**

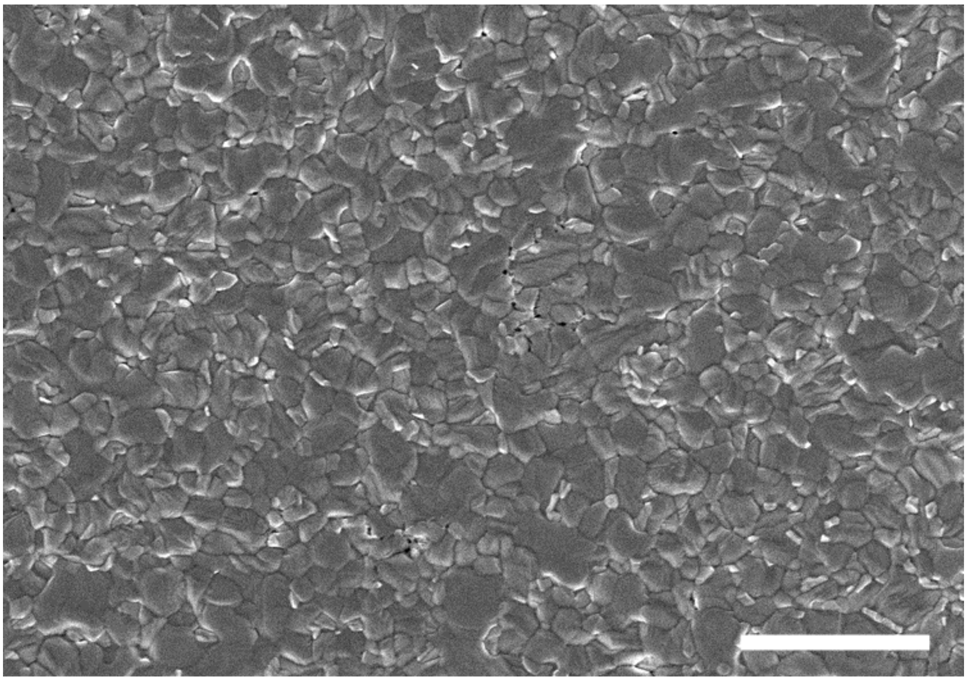

Figure S2. Low-magnification SEM image of GBL:MSM perovskite film

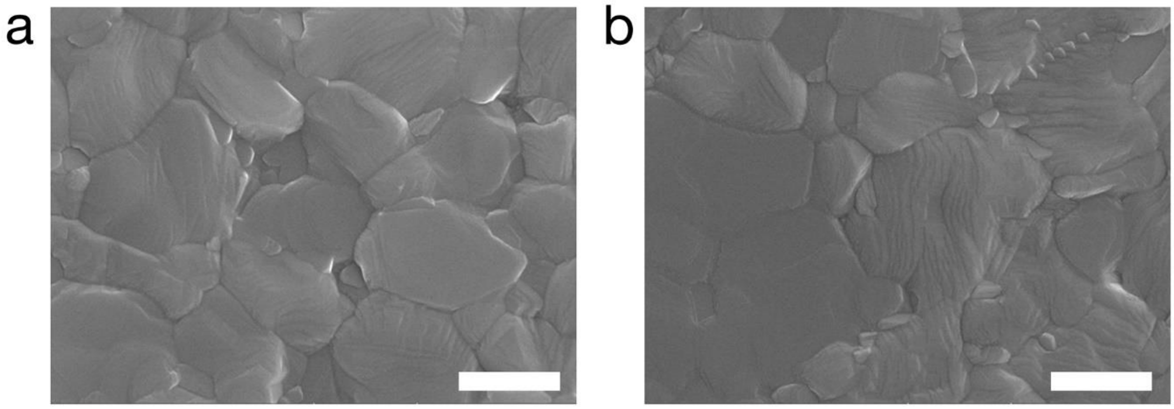

Figure S3. SEM Images of perovskite film fabricated by a) dripping and b) bathing of BA antisolvents. The white scale bar is 1 μm.

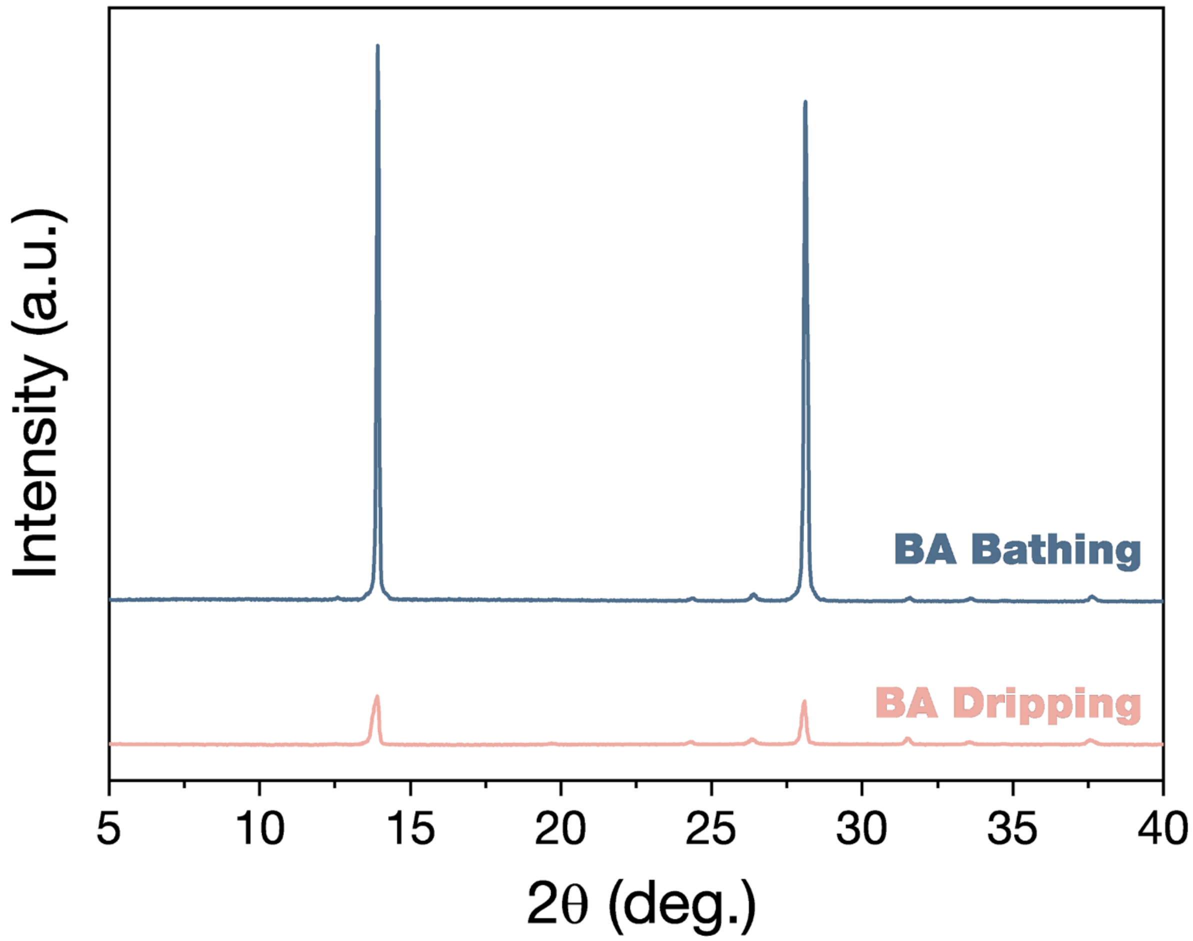

Figure S4. XRD spectra of perovskite film fabricated by dripping of and bathing in BA antisolvent.

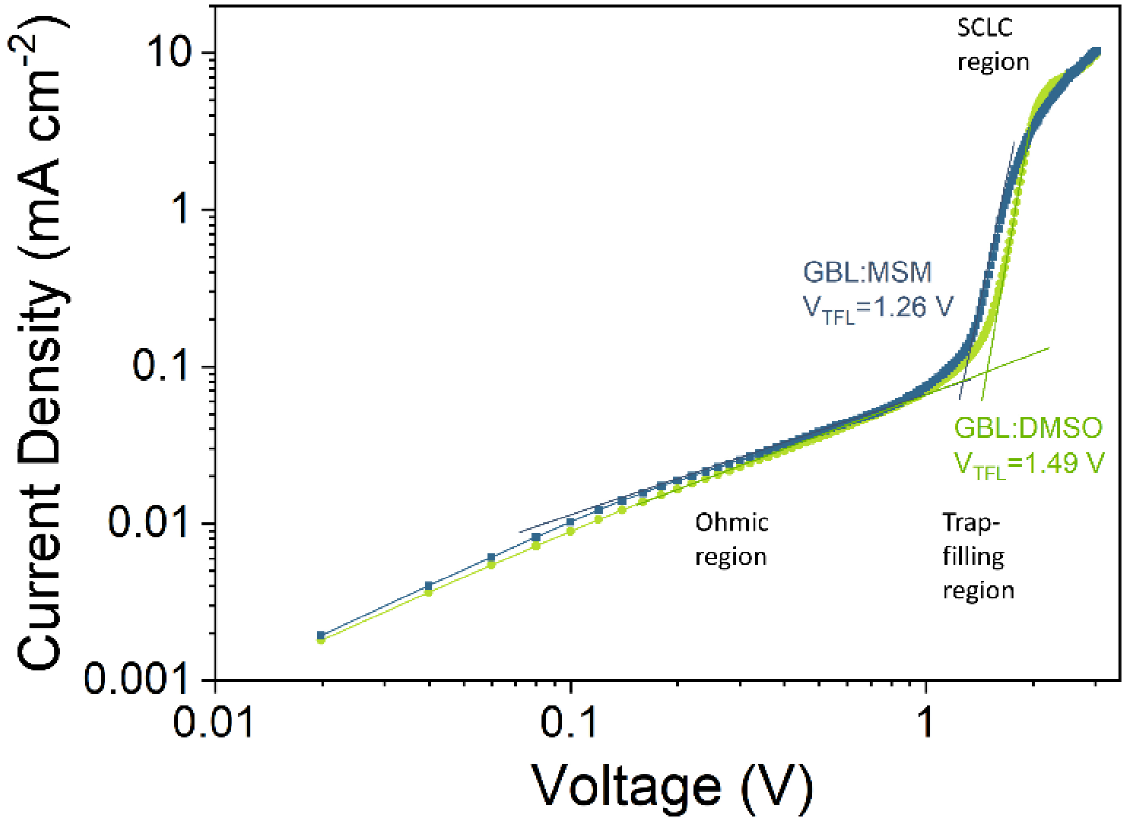

Figure S5. J-V curves for hole-only devices.

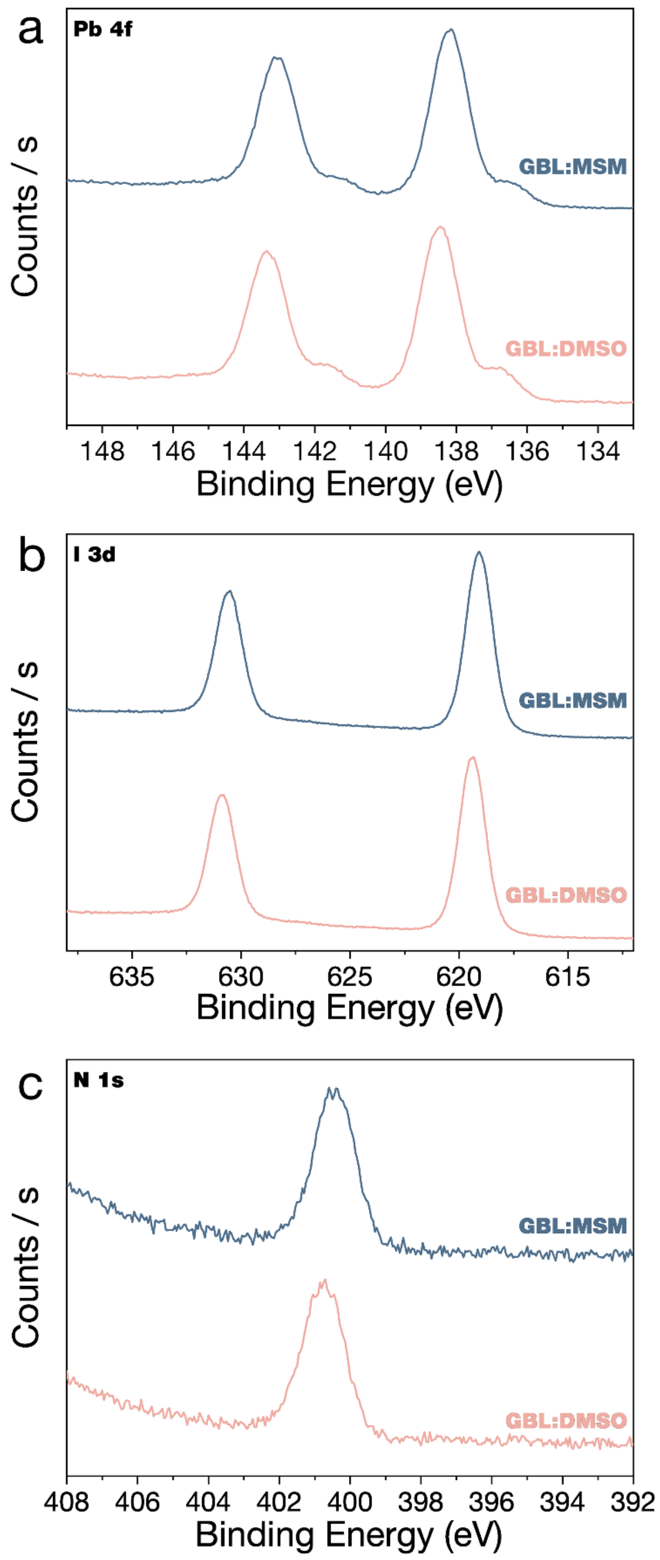

Figure S6. XPS spectra of perovskite films fabricated by using GBL:MSM and GBL:DMSO solvents.

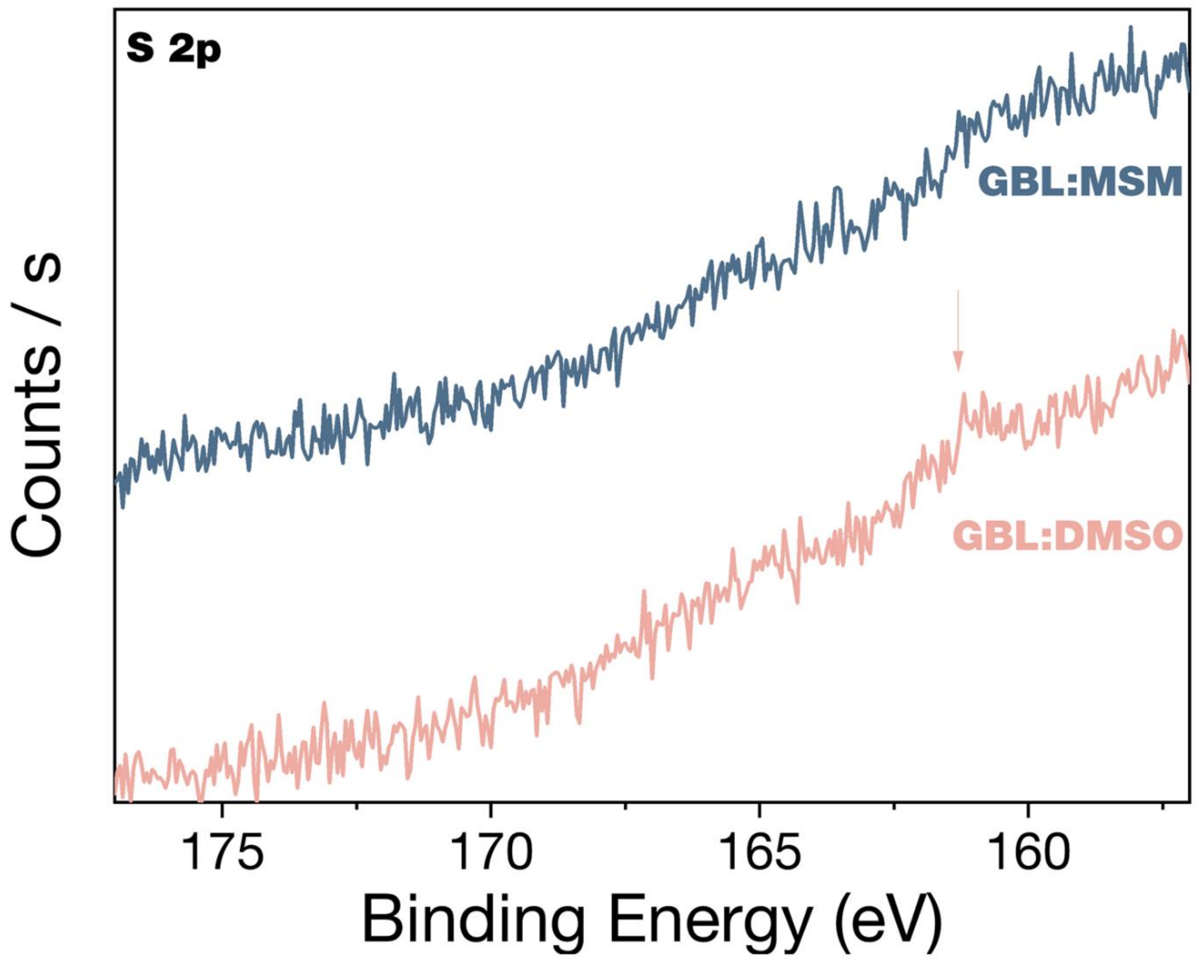

Figure S7. XPS S 2p spectra of perovskite films made by using GBL:MSM and GBL:DMSO.

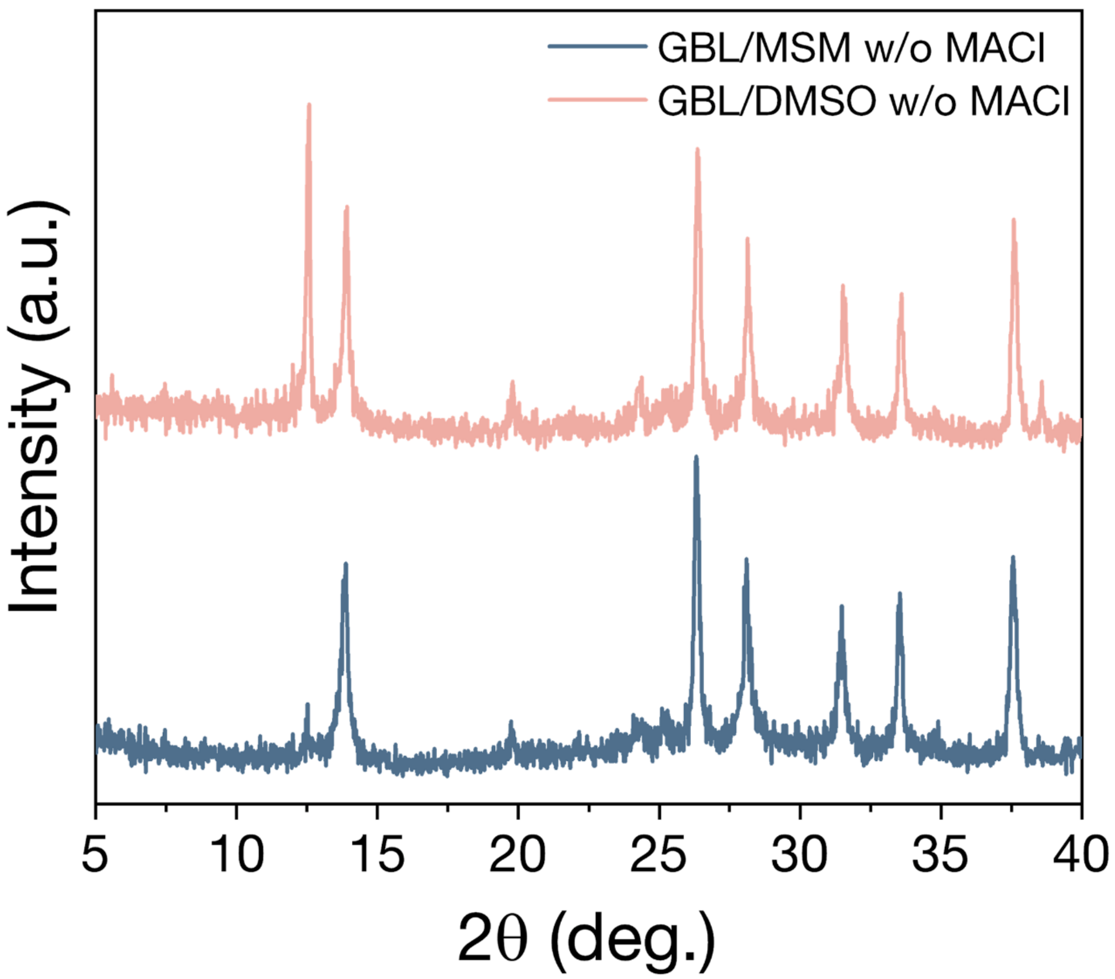

Figure S8. XRD spectra of perovskite films fabricated by using GBL:MSM and GBL:DMSO solvents, without containing MACl additive in precursor solution.

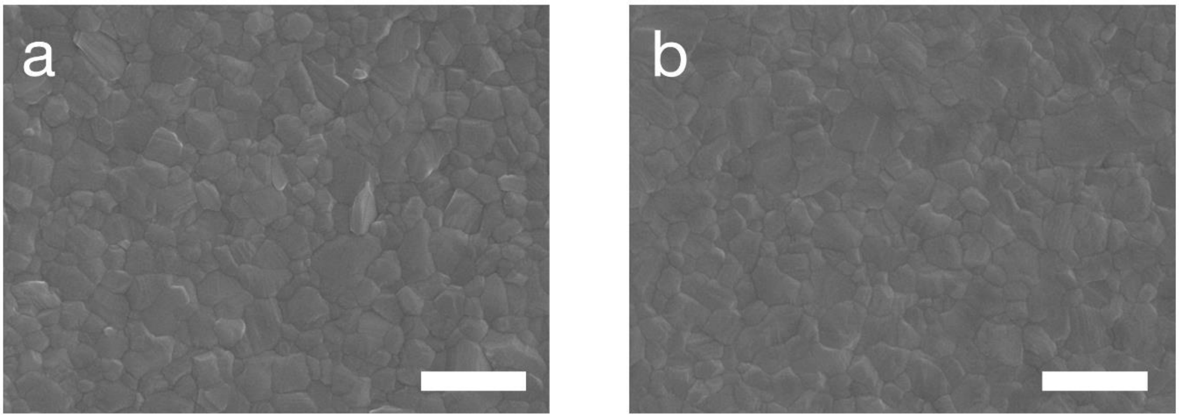

Figure S9. SEM images of perovskite films fabricated by using GBL:MSM and GBL:DMSO solvents, without MACl additive in precursor solution. Scale bar is 1 μm.

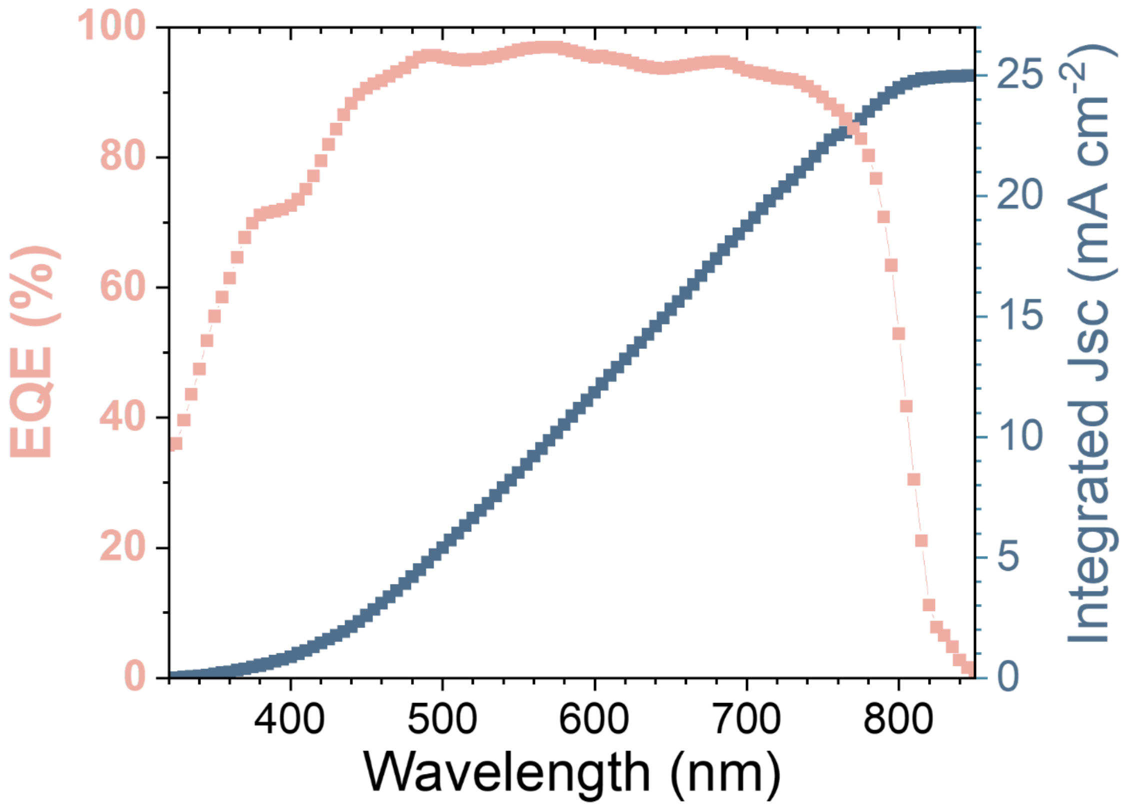

Figure S10. External quantum efficiency and integrated J_{SC} curves of the representative device fabricated by using GBL:MSM.

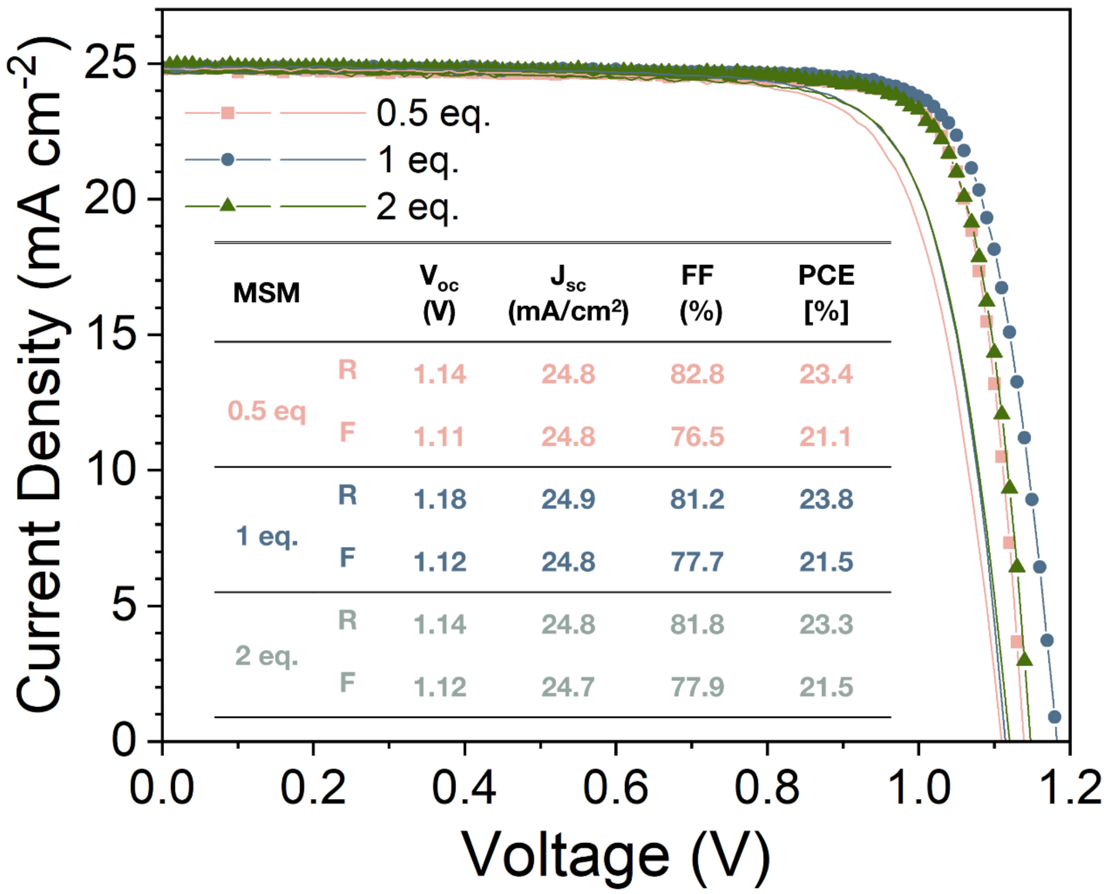

MSM		V_{oc} (V)	J_{sc} (mA/cm^2)	FF (%)	PCE [%]
0.5 eq	R	1.14	24.8	82.8	23.4
	F	1.11	24.8	76.5	21.1
1 eq.	R	1.18	24.9	81.2	23.8
	F	1.12	24.8	77.7	21.5
2 eq.	R	1.14	24.8	81.8	23.3
	F	1.12	24.7	77.9	21.5

Figure S11. J-V Curves of PSCs with varying amount of MSM in the precursor solution.

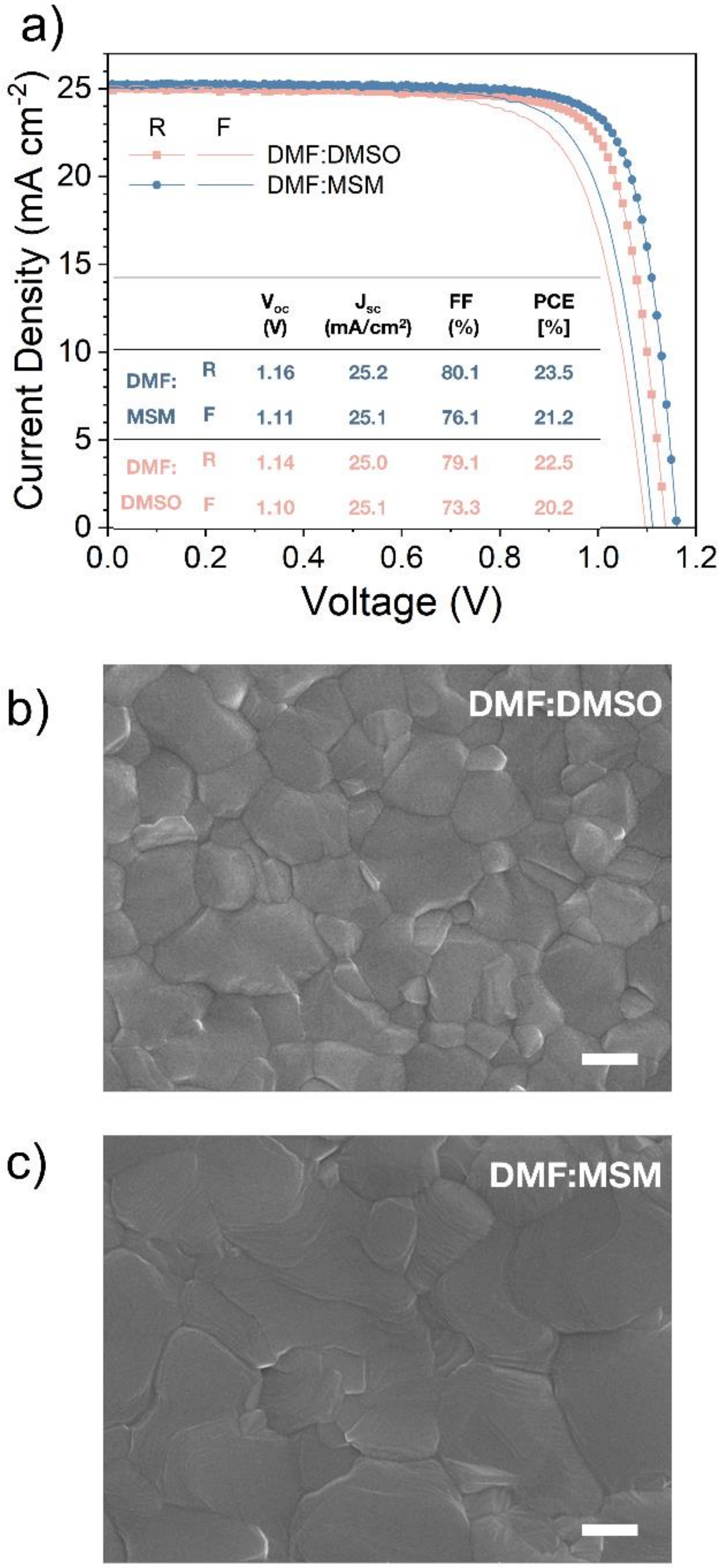

Figure S12. a) J-V curves of the PSCs made by using DMF:MSM and DMF:DMSO. The SEM images of perovskite films made by using b) DMF:DMSO and c) DMF:MSM solvent system. Scale bar is 500 nm.

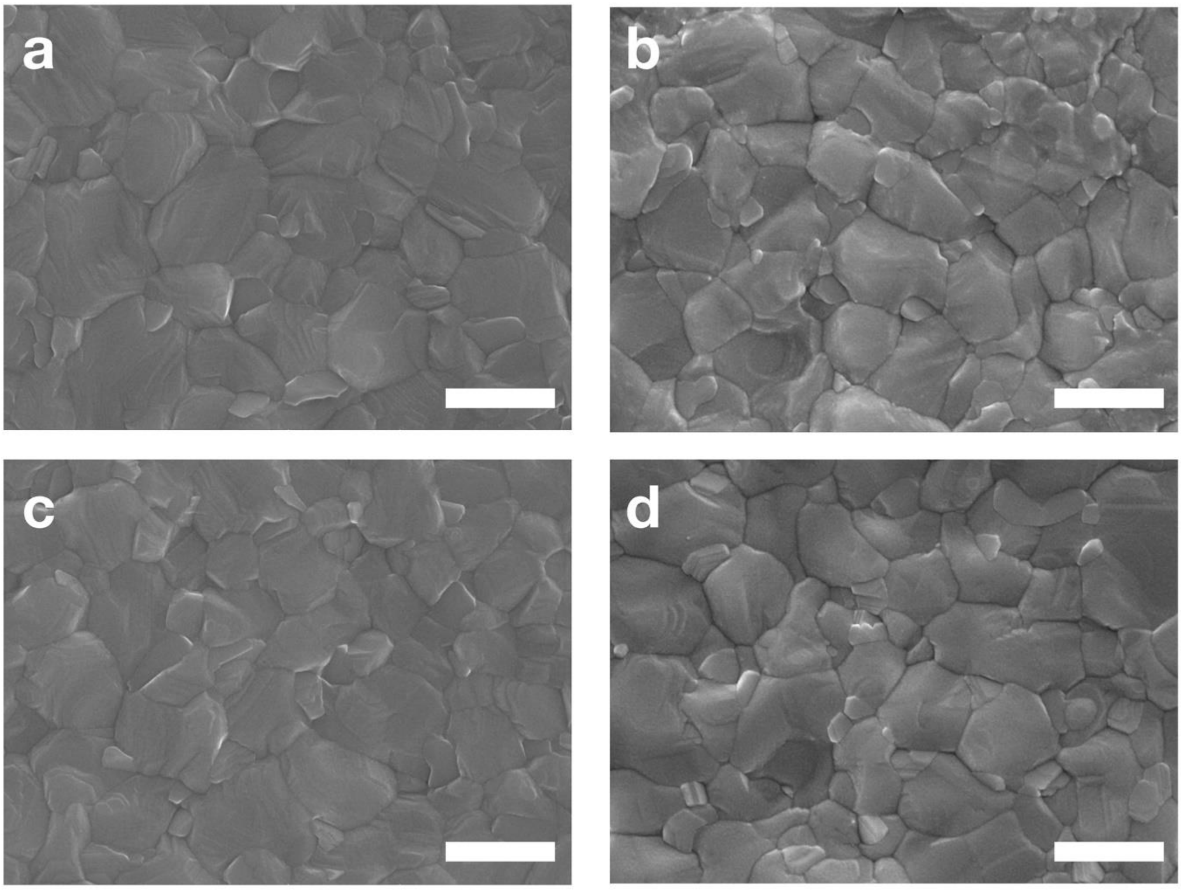

Figure S13. SEM images of perovskite films. Solvents and relative humidity conditions for perovskite films are a) GBL:DMSO (25% RH), b) GBL:DMSO (60% RH), c) GBL:MSM (25% RH), and d) GBL:MSM (60% RH), respectively. All the scale bars are 1 μm.

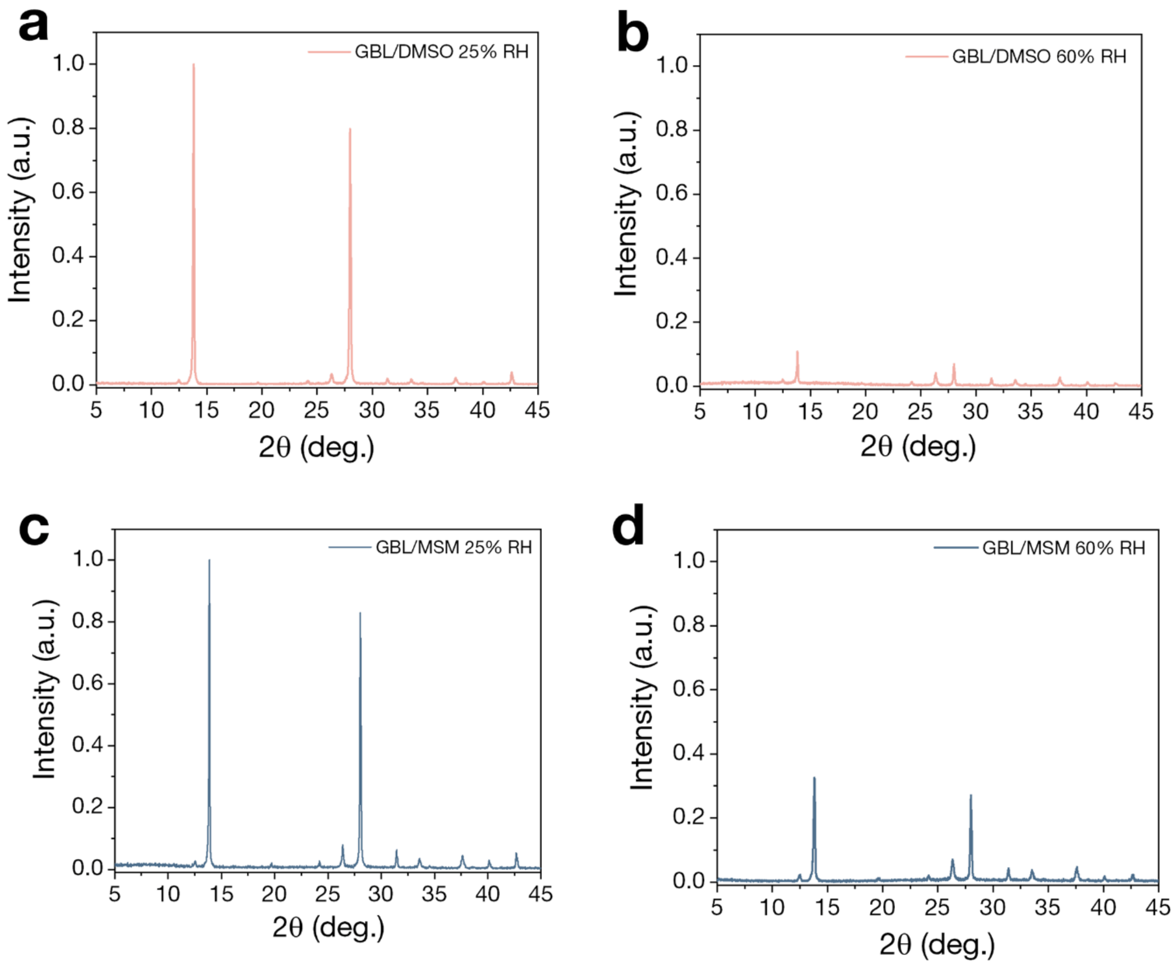

Figure S14. XRD spectra of perovskite films with different processing solvents and conditions. a) GBL:DMSO 25% RH, b) GBL:DMSO 60% RH, c) GBL:MSM 25% RH, and d) GBL:MSM 60% RH, respectively.

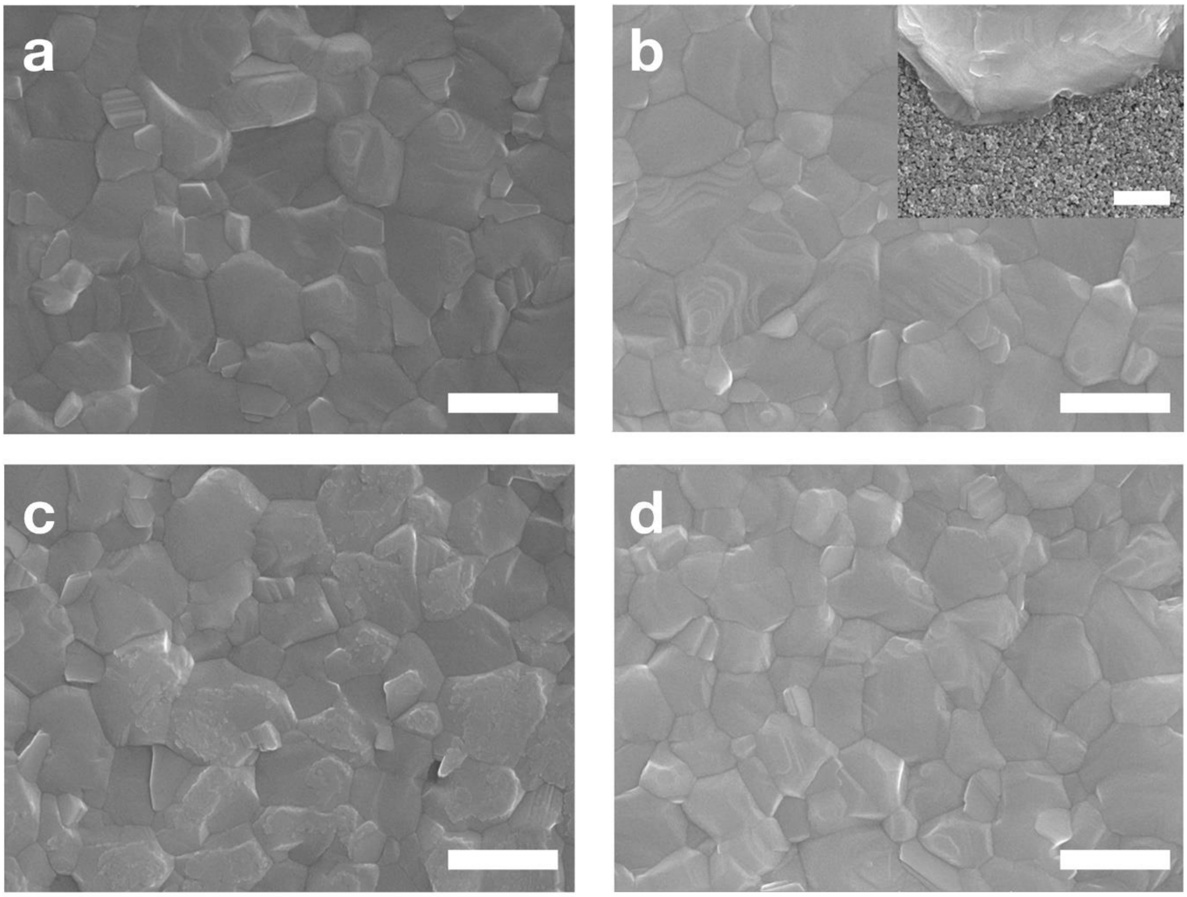

Figure S15. SEM images of perovskite films with different processing solvents and temperatures. a) GBL:DMSO (24 °C), b) GBL:DMSO (10 °C), c) GBL:MSM (24 °C), and d) GBL:MSM (10 °C), respectively. All the scale bars are 1 μm.

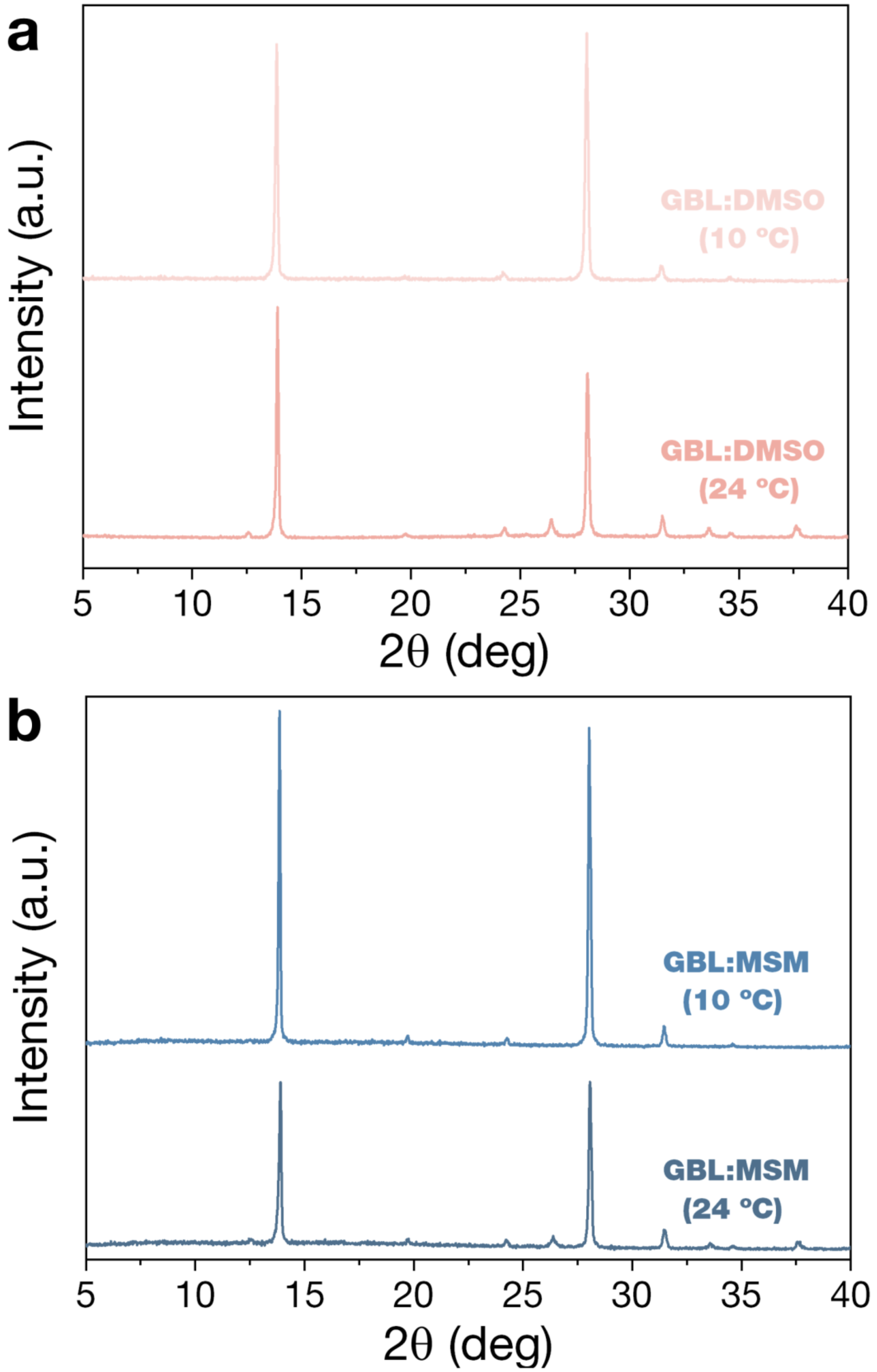

Figure S16. XRD spectra of perovskite films fabricated by using a) GBL:DMSO and b) GBL:MSM under different processing temperature.

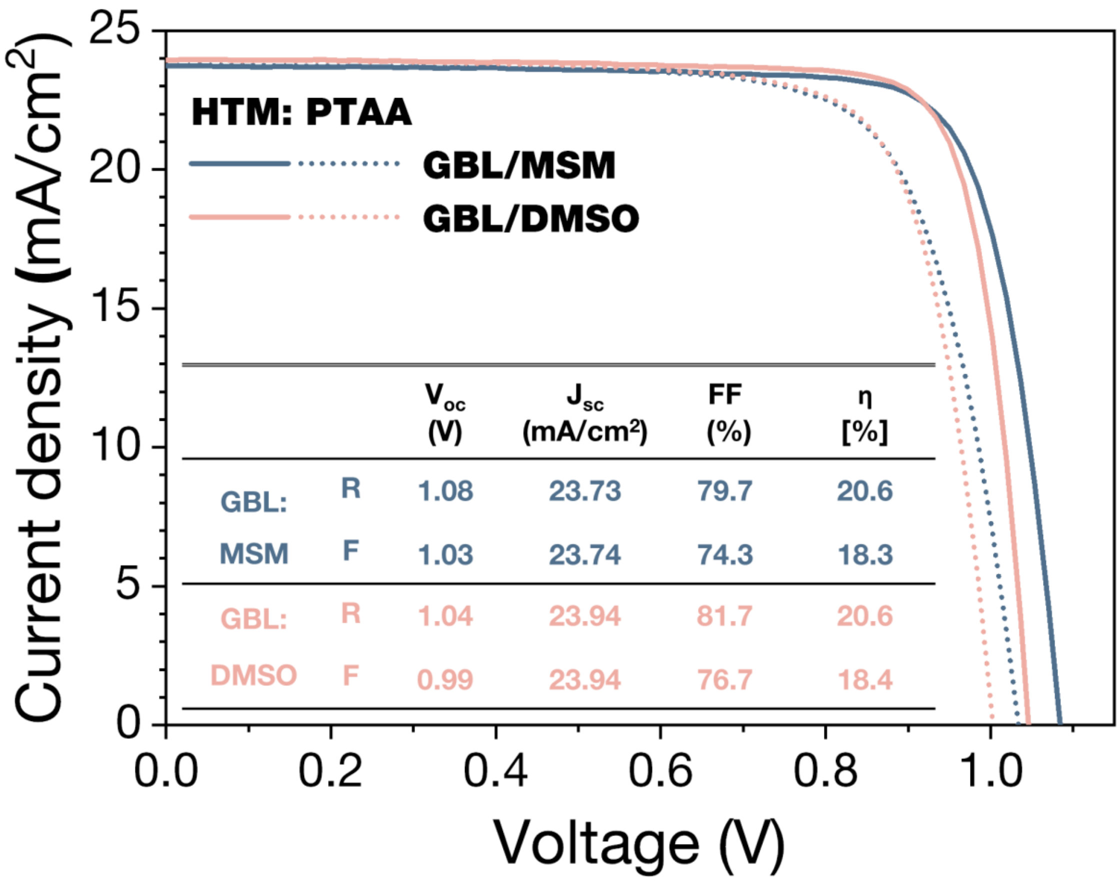

Figure S17. J-V curves of PSCs fabricated by using GBL:MSM and GBL:DMSO solvents and PTAA as a HTM. (inset) A table represents solar parameters of devices.

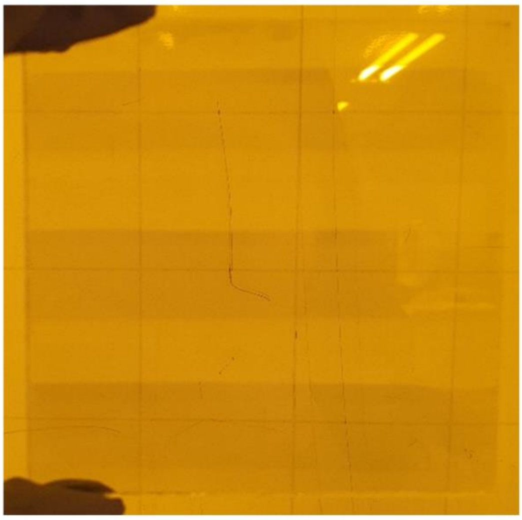

Figure S18. An optical image of glass substrate with patterned ITO for the fabrication of devices from the same substrate.

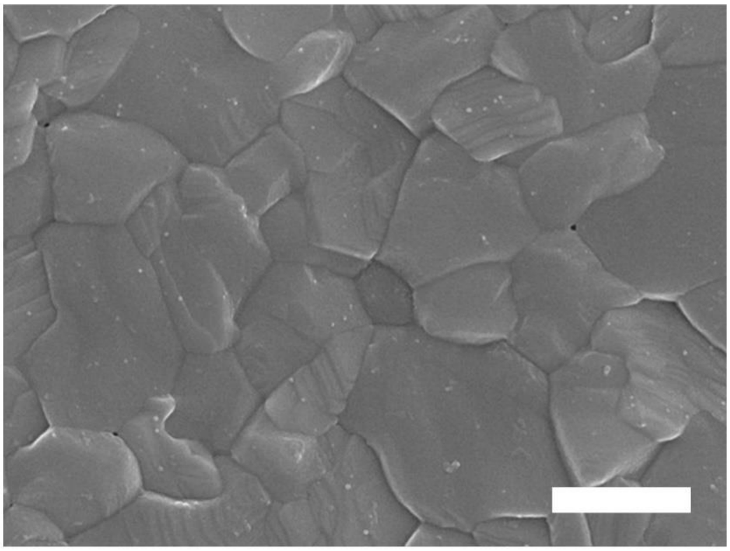

Figure S19. A SEM image of perovskite film made by using GBL:MSM solvents in 7×7 cm^2 substrate.

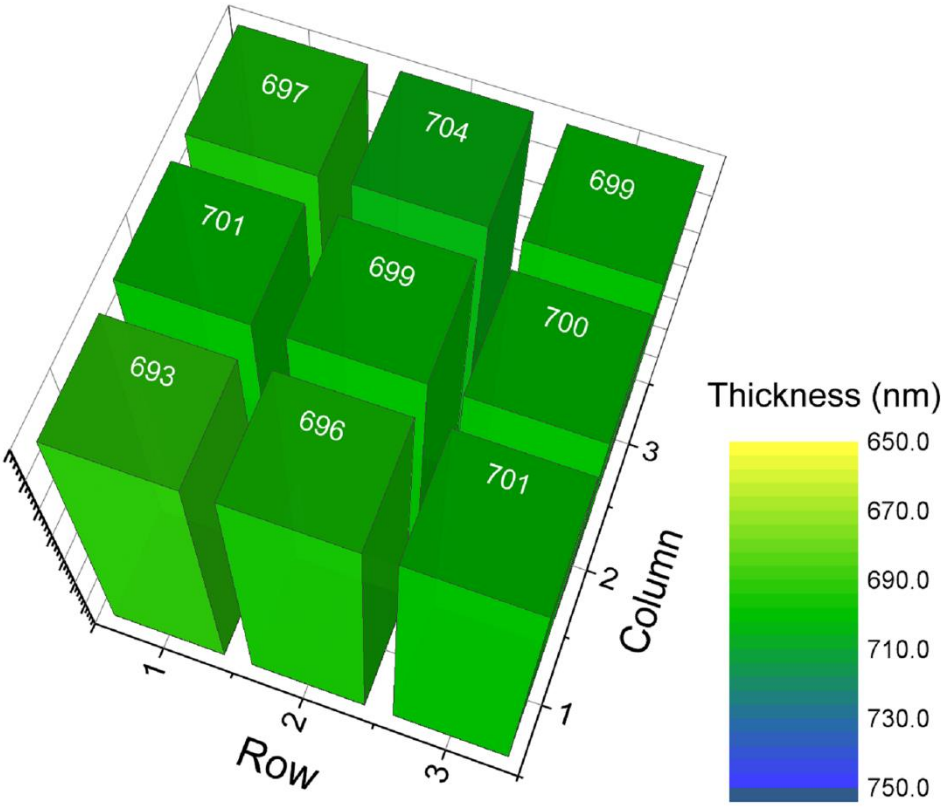

Figure S20. Thickness values of the perovskite film measured in a different position in the same, 7×7 cm^2 substrate.
